# Early antibiotic exposure and development of asthma and allergic rhinitis in childhood

**DOI:** 10.1186/s12887-019-1594-4

**Published:** 2019-07-05

**Authors:** Jeffrey Ni, Hannah Friedman, Bridget C. Boyd, Andrew McGurn, Piotr Babinski, Talar Markossian, Lara R. Dugas

**Affiliations:** Loyola University Medical Center, 2160 S 1st Ave, Maywood, IL 60153 USA

## Abstract

**Background:**

The prevalence of pediatric allergic diseases has increased rapidly in the United States over the past few decades. Recent studies suggest an association between the increase in allergic disease and early disturbances to the gut microbiome. The gut microbiome is a set of intestinal microorganisms that begins to form during birth and is highly susceptible to disturbance during the first year of life. Early antibiotic exposure may negatively impact the gut microbiota by altering the bacterial composition and causing dysbiosis, thus increasing the risk for developing childhood allergic disease.

**Methods:**

We performed a retrospective chart review of data in Loyola University Medical Center’s (LUMC) Epic system from 2007 to 2016. We defined antibiotic exposure as orders in both the outpatient and inpatient settings. Inclusion criteria were being born at LUMC with at least two follow up visits. Asthma and allergic rhinitis diagnoses were obtained using ICD 9 and ICD 10 codes. We controlled for multiple confounding factors. Using Stata, bivariate logistic regression was performed between antibiotics from 0 to 12 months of life and development of disease. This analysis was repeated for total lifetime antibiotics. We defined statistically significant as *p* < .05.

**Results:**

The administration of antibiotics within the first 12 months of life was significantly associated with lifetime asthma (OR 2.66; C. I 1.11–6.40) but not allergic rhinitis. There was a significant association between lifetime antibiotics and asthma (OR 3.54; C. I 1.99–6.30) and allergic rhinitis (OR 2.43; C. I 1.43–4.11).

**Conclusion:**

Antibiotic administration in the first year of life and throughout lifetime is significantly associated with developing asthma and allergic rhinitis. These results provide support for a conservative approach regarding antibiotic use in early childhood.

## Background

Overuse of antibiotics is a growing public health concern. While in the last two decades outpatient antibiotic prescriptions have decreased significantly, inpatient broad spectrum antibiotic use has continued to rise [1–3]. In fact, despite efforts to promote conservative antibiotic stewardship in the United States, antibiotics are still the most frequently dispensed outpatient prescription medication, accounting for approximately 25% of all pediatric medication prescriptions [3]. Notably, five out of the top six prescribed medications for the children in the United States are antibiotics, with Amoxicillin and Azithromycin being the most common [3]. A large study investigating bacterial prevalence and antibiotic prescribing trends for pediatric acute respiratory tract infections (ARTIs), estimated that about 30% of antibiotic prescriptions are unnecessary [4, 5]. Consequently, there are about 11.5 million antibiotics prescribed annually for illnesses in which a bacterial pathogen is not the expected etiology of the illness, and thus antibiotics are not warranted [4]. Though antibiotics are an important part of modern healthcare, there are some potential adverse effects of which to be aware, including unwanted side effects, antibiotic resistance, and alteration of the gut microbiota. In particular, the gut microbiome hypothesis has recently emerged as a link between antibiotic exposure and disease development. It has been suggested that the relationship between early antibiotic exposure and dysbiosis of the gut microbiota may have significant implications for the health of children now and as they grow into adults.

The gut microbiota is comprised of trillions of microbes in the human intestinal tract, and contains over a thousand of different species of bacteria [6]. Previous studies have suggested that the first year of life represents a critical period of development and that by about three years of age, the microbiota is fully mature [7, 8]. The gut microbiota has also been demonstrated to play an important role in the human immune sys and maintenance of homeostasis. Alterations in the gut microbiota are a purported mechanism underlying the “hygiene hypothesis” [9], in which children who are exposed to a wide range of environmental and nutritional factors that promote a diverse and robust microbiota are less prone to atopy and asthma. In fact, gut dysbiosis has been linked to early disruptions in the regulation of the immune system [10], and thus to the development of chronic atopic and inflammatory respiratory diseases such as asthma and allergic rhinitis [11–13]. Additionally, according to the Center for Disease Control (CDC), the prevalence of these diseases in the United States has continued to rise in the past two decades despite significant medical advancements [14]. New evidence suggests that there may be a connection between early antibiotic exposure altering the development of the gut microbiota, and subsequently the immune system, increasing the risk for developing the aforementioned diseases [15, 16]. However, relatively few studies have investigated the effects of timing of antibiotic exposure on future health outcomes, and whether there is a period during early development when the gut microbiota are most susceptible to gut dysbiosis. In addition, few studies have examined the relationship between increasing antibiotic doses and subsequent effects on propensity for disease development in a dose response relationship. Our study aims to investigate this temporal relationship, as well as the effects of early antibiotic exposure on the future propensity for disease development later in childhood. Consistent with the gut microbiome hypothesis, we hypothesize that children exposed to antibiotics during the first year of life will be more likely to be diagnosed with asthma or allergic rhinitis later in childhood, compared to children not receiving antibiotics during their first year of life. We also hypothesize that this relationship will present in a dose-dependent manner, with higher doses of antibiotics leading to increasing propensity to develop disease outcomes.

## Methods

### Study design

We conducted a retrospective cohort study using electronic medical record (EMR) data from 2007 to 2016 at a large academic health institution. A single person completed data extraction and coding of variables for this study. Inpatient, emergency room, immediate care, and outpatient clinic encounters at the institution were included in the study. Children ages 1–10 years were included in this study; children younger than 1 year of age at the time of our study were excluded from the sample due to low number of diagnoses due to age. All children included were born at and attended at least two subsequent visits at this institution. Our dataset contained birth information on sex, age, race/ethnicity, zip code, birth weight, gestational age, admission to the neonatal intensive care unit (NICU vs. normal nursery), and method of delivery. For each additional visit, our data also contained recorded height, weight, and any current or past diagnoses. Primary outcomes included childhood asthma and allergic rhinitis. Children with missing data were excluded from the study. The study was approved by Loyola University Chicago institutional review board (IRB) and marked exempt.

### Study exposure

Antibiotic exposure was defined as a physician order for outpatient or administration of inpatient oral antibiotics or intravenous antibiotics. Children received at least one of the following antibiotics in this study: Penicillin, Amoxicillin, Gentamicin, Vancomycin, Clindamycin, Sulfamethoxazole/Trimethoprim, Cephalexin, Ampicillin, Cefotaxime, Ceftriaxone, Azithromycin, Cefdinir and Ceftazidime. We studied two exposures: our first exposure maintained antibiotic exposure as a continuous variable in terms of dosages, and our second exposure created binary exposure groups, categorizing antibiotics as receiving at least one order or administration vs. receiving no orders or administrations within a designated time frame. First, we compared children who received at least one dose of antibiotics in the first year of life to children who were not exposed during this time. We also compared children who received at least one dose of antibiotics in their lifetime to children who never received antibiotics. Lastly, we examined the dose-response relationship using an ordinal logistic regression analysis of each additional antibiotic prescription using the continuous antibiotic exposure group, and compared outcomes between first year and lifetime antibiotic exposure and the development of our primary disease outcomes.

### Study outcomes

Our primary disease outcomes were asthma and allergic rhinitis. All diseases except for obesity were diagnosed based on their respective *International Classification of Diseases, Ninth Revision,* and *International Classification of Diseases, Tenth Revision* codes (Table 1). All subclassifications of asthma, including intermittent and mild, moderate and severe persistent asthma, were also obtained using ICD coding (Table 1).

**Table 1:** ICD-9 and ICD-10 Codes Used for Disease Identification

Disease	ICD-9 Code	ICD-10 Code
Asthma	493.00 Extrinsic asthma, unspecified	J45.2-Mild intermittent asthma
	493.01 Extrinsic asthma with status asthmaticus	J45.3-Mild persistent asthma
	493.02 Extrinsic asthma with exacerbation	J45.4-Moderate persistent asthma
	493.10 Intrinsic asthma, unspecified	J45.5-Severe persistent asthma
	493.20 Chronic obstructive asthma, unspecified	J45.9-Other and unspecified asthma
	493.81 Exercise induced bronchospasm	
	493.82 Cough variant asthma	
	493.90 Unspecified asthma	
Allergic Rhinitis	477 Allergic rhinitis	J30.0-Vasomotor rhinitis
	477. Allergic rhinitis	J30.1-Allergic rhinitis due to pollen
	477.0 Allergic rhinitis due to pollen	J30.2-Other seasonal allergic rhinitis
	477.1 Allergic rhinitis due to food	J30.5-Allergic rhinitis due to food
	477.2 Allergic rhinitis due to animal (cat) (dog) hair and dander	J30.8-Other allergic rhinitis
	477.8 Allergic rhinitis due to other allergen	J30.9-Allergic rhinitis, unspecified
	477.9 Allergic rhinitis, cause unspecified	

### Covariates

The following covariates were adjusted for in the multi-level analysis: race/ethnicity (Non-Hispanic (NH) white, NH black, Hispanic, and NH other), age, sex (male vs. female), delivery method (caesarean section vs. vaginal), prematurity (< 37 weeks gestation age), birth weight, NICU admission status, and socio-economic status (SES). We categorized birth weight into low birthweight (< 5.5 lbs), normal birthweight (5.5–8.8 lbs), and high birthweight (> 8.8 lbs) at the time of birth. SES was determined based on zip code and poverty levels from CDC U. S Census data from the year 2000 [17]. Based on this data, we categorized SES into three groups based on percentage of households within each zip code area living below the poverty threshold: < 10% poverty, 10–20% poverty, and > 20% poverty.

### Data analyses

Data are presented as means ± standard errors (SE), and proportions (%). Analyses were performed in Stata/SE Version 12.0. Multivariable analyses were conducted using multiple binomial logistic regression models. Confounding variables were controlled for in the models, yielding adjusted odds ratios. We used a 95% confidence interval and defined statistical significance as *p* < 0.05.

## Results

### Study participants

**Fig. 1:**
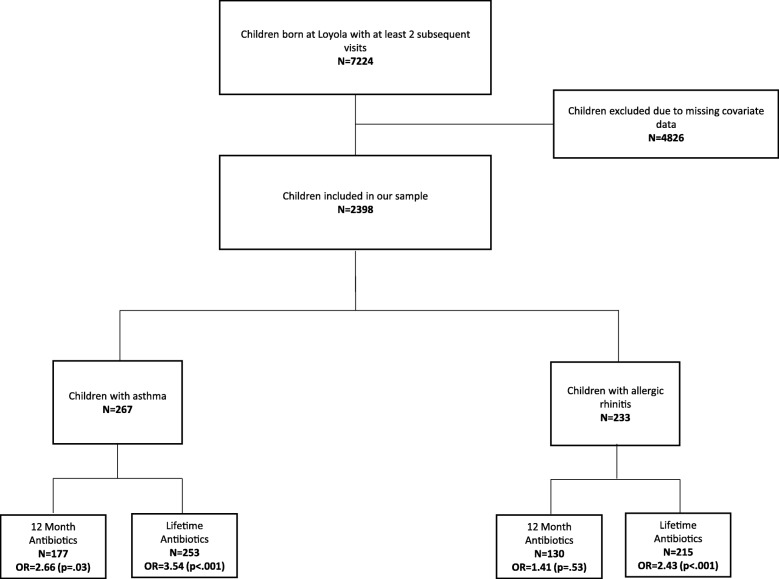
Title: Study Criteria Flowsheet and Disease Sample Sizes with Antibiotic Administration. Legend: Our original sample size consisted of a total of 7224 children. We excluded 4826 children from our analysis due to missing covariate data. Our final sample size was 2398 children. In this sample, antibiotic usage was associated with asthma and allergic rhinitis

In our sample, there were a total of 7224 children born at the institution from 2007 to 2016 who received at least two subsequent visits at the health center (Table 2). Our study sample was limited by missing covariate data on gestational age at birth and delivery method, thus narrowing our study sample to 2398 children (Fig. 1). The mean age at the time of our study was 5.7 ± .05 years with a maximum age of 9 years, and 51.0% was male (Table 2). Overall, 11.0% of our sample had asthma, and 9.7% had allergic rhinitis.

**Table 2:** Sample Demographics and Disease Prevalence

	*N* = 2398	
	Mean	SE
Age (years)	5.7	0.05
Gender		
Male %	51	0.01
Female %	48.9	0.01
Race		
White %	37.9	0.01
Black %	20.8	0.01
Hispanic %	31.7	0.01
Other %	9.4	0.01
Birth		
C-Section %	40.7	0.01
Preterm (< 37 weeks) %	18.5	0.01
NICU %	18	0.01
High Birthweight % (> 8.8 lbs)	10.1	0.01
Low Birthweight % (< 5.5 lbs)	13.3	0.01
Living area with poverty		
< 10% Poverty %	17.3	0.01
10–20% Poverty %	53.3	0.01
> 20% Poverty %	29.3	0.01
Disease Status		
Asthma %	11.1	0.01
Allergic Rhinitis %	9.7	0.01
Eczema %	15.4	0.01
Obesity %	9.7	0.01

### Difference in antibiotic exposure

In our sample, 44.2% of children were exposed to antibiotics within the first year of life, and 73.2% over their lifetime. Total lifetime antibiotic exposures, captured as antibiotic courses prescribed or ordered, ranged from 0 to 59 over the measurement period; amongst children who received antibiotics, the average number of exposures in the first year of life was 1.6 ± .07 courses of antibiotics, and the average number of lifetime exposures was 4.4 ± .12 courses of antibiotics. Overall, females were less likely to receive antibiotics in their lifetime compared to their male counterparts (OR 0.78; 95% CI 0.64–0.94). In addition, NH black children (OR 0.52; 95% CI 0.40–0.69), Hispanic children (OR 0.59; 95% CI 0.47–0.76) and other NH children (OR 0.63; 95% CI 0.44–0.88) were also less likely to receive antibiotics compared to NH white children. These racial and ethnic differences are consistent with previous research [18]. Compared to children born vaginally, at term, and without admission to the NICU, children born by C-section (OR 1.26; 95% C. I 1.04–1.54), prematurely (OR 2.05; 95% C. I 1.33–3.17) and with an admission to the NICU (OR 6.66; 95% C. I 3.89–11.41) were significantly more likely to receive antibiotics throughout life (Table 3).

**Table 3:** Demographic influence on antibiotic administration throughout lifetime and within the first year of life

	12 Months Exposure			Lifetime Exposure		
	Odds Ratio	*P*-Value	95% C.I	Odds Ratio	*P*-Value	95% C.I
Gender						
Male	Referent					
Female	0.83	0.05	0.69–1.00	0.78	0.01	0.64–0.94
Race						
White	Referent					
Black	0.68	0.00	0.52–0.89	0.52	0.00	0.40–0.69
Hispanic	0.60	0.00	0.38–0.75	0.59	0.00	0.47–0.76
Other	0.89	0.48	0.64–1.23	0.63	0.01	0.44–0.88
Birth						
Vaginal	Referent					
C-section	1.07	0.48	0.89–1.30	1.26	0.02	1.04–1.54
Term	Referent					
Preterm (< 37 Weeks)	1.92	0.00	1.36–2.73	2.05	0.00	1.33–3.17
Non-NICU	Referent					
NICU	11.77	0.00	7.95–17.41	6.66	0.00	3.89–11.41
Normal Birthweight	Referent					
High Birthweight (> 8.8 lbs)	1.13	0.59	0.73–1.75	0.90	0.69	0.55–1.48
Low Birthweight (< 5.5 lbs)	1.21	0.20	0.91–1.61	1.08	0.62	0.79–1.49
Poverty Status						
< 10% Poverty	Referent					
10–20% Poverty	1.17	0.22	0.95–1.58	1.09	0.83	0.83–1.43
> 20% Poverty	1.00	0.99	0.79–1.41	0.86	0.64	0.64–1.16

### Relationship between antibiotics and disease

Exposure to antibiotics within the first year of life was significantly associated with asthma (OR 2.66; 95% C. I 1.11–6.40), but not with allergic rhinitis (OR 1.41; 95% C. I 0.48–4.14). Furthermore, there was a significant association between lifetime antibiotic exposure and asthma (OR 3.54; 95% C. I 1.99–6.30) and allergic rhinitis (OR 2.43; 95% C. I 1.43–4.11) (Table 4). Lastly, in children who received antibiotics in the first year of life, there was a significant antibiotic dose-response relationship with the future development of asthma (OR 1.18; 95% C. I 1.02–1.38). We also found a significant dose dependence in the association between lifetime antibiotic administration and the eventual development of asthma (OR 1.09; 95% C. I 1.07–1.11) and allergic rhinitis (OR 1.06; 95% C. I 1.04–1.09) (Table 5).

**Table 4:** Antibiotic Administration Correlated with Asthma and Allergic Rhinitis reported as odds ratios (OR). Adjusted odds ratio (aOR) controlled for covariates including age, sex, race/ethnicity, socioeconomic status, delivery method, NICU status, birthweight, and prematurity

	Asthma						Allergic Rhinitis					
	OR	*P*-value	95% C.I	aOR	*P*-value	95% C.I	OR	*P*-value	95% C.I	aOR	*P*-value	95% C.I
0–12 Months	3.57	<.001	1.79–7.13	2.66	0.03	1.11–6.40	1.66	0.17	.80–3.43	1.41	0.53	0.48–4.14
Lifetime	7.57	<.001	4.38–13.07	3.54	0.00	1.99–6.30	4.85	<.001	2.97–7.91	2.43	<.001	1.43–4.11

**Table 5:** Number of antibiotic orders in the first year of life and throughout life correlated with asthma and allergic rhinitis reported odds ratios (OR). Adjusted odds ratio (aOR) controlled for covariates including age, sex, race/ethnicity, socioeconomic status, delivery method, NICU status, birthweight, and prematurity.

	Asthma						Allergic Rhinitis					
	OR	*P*-value	95% C.I	aOR	*P*-value	95% C.I	OR	*P*-value	95% C.I	aOR	*P*-value	95% C.I
0–12 Months	1.14	<.001	1.10–1.17	1.18	0.03	1.02–1.38	1.04	0.01	1.01–1.07	0.91	0.56	0.66–1.25
Lifetime	1.11	<.001	1.09–1.13	1.09	<.001	1.07–1.11	1.09	<.001	1.07–1.10	1.06	<.001	1.04–1.09

## Discussion

Our disease prevalence rates were comparable to national data for asthma and allergic rhinitis [19, 20]. Consistent with our hypothesis, children exposed to antibiotics throughout the first year of life were more likely to have a diagnosis of asthma when compared to children who had did not receive antibiotics in the first year of life. These results suggest that the first year of life may be an especially sensitive time for the development of asthma when an antibiotic insult is inflicted upon the developing gut microbiota. We also found a significant positive relationship between lifetime antibiotic exposure and future likelihood to have a diagnosis for asthma and allergic rhinitis compared to children who had never been exposed to antibiotics. These adjusted odds ratios were greater than the one observed in children receiving antibiotics in the first year, indicating that although the gut microbiota may stabilize and mature by the first year of life, it may still be sensitive to insult as the child grows, or that the insults may be cumulative and irreversible. In addition, we observed a significant dose response relationship in both the associations between antibiotics in the first year of life and the development of asthma, and between lifetime antibiotics and the development of asthma and allergic rhinitis. This relationship suggests that antibiotic insult to the gut microbiota may be additive, such that the more a child is exposed to antibiotics, the greater their likelihood to develop disease in childhood. This is consistent with our hypothesis that repeated antibiotics can exacerbate microbiota dysbiosis [15, 16].

Contrary to our hypothesis, we did not find a significant positive association between antibiotic exposure in the first year of life and development of allergic rhinitis. Relatively few studies have examined the relationship between antibiotics within the first year of life and allergic rhinitis; however, previous studies in different countries have indicated a weak, positive relationship between antibiotic exposure in the early stages of life and allergic rhinitis [21, 22]. Our study results may have been limited by smaller sample size from a single institution, and inability to distinguish between different antibiotic classes.

In terms of allergic rhinitis, the gut microbiota is emerging as a novel target for early intervention in the setting of rising pediatric atopic diseases. Dysbiosis in the gut microbiota has previously been correlated with allergic diseases, and past research has suggested that the gut microbiota is most sensitive to change during the first year of development. However, varying conclusions have been made regarding the association between antibiotic exposure and development of allergic diseases [23–26]. Recent studies have suggested that a higher bacterial ratio between *Klebsiella*, an opportunistic pathogen, and *Bifidobacterium*, a commensal inhabitant of the gut microbiota, may predispose to allergic diseases [27]. In support of this, further studies have indicated that the administration of infant probiotics may alter this ratio favorably and be protective against the future development of allergic disease [28]. The effects also seem to be long-term, as previous research has demonstrated incomplete recovery of the gut microbiome and decreased microbiota diversity after antibiotic administration [29]. While our study did not find a significant correlation between first year antibiotics and allergic rhinitis, we did find a correlation between first year antibiotics and asthma which is frequently associated with allergic rhinitis [30]. Thus, it is plausible that a correlation does exist between early antibiotics and allergic rhinitis which our study did not identify. Furthermore, it is also a possibility that the first year of life is not as sensitive for antibiotics increasing the risk for developing allergic rhinitis, and that a more chronic temporal relationship exists, as we found both a significant overall and dose response relationship between lifetime antibiotics and allergic rhinitis. Further studies are required to explore this timeline.

There are several limitations to our study. First, we cannot exclude reverse causality as a reason for the positive association that we found between lifetime antibiotic exposure and asthma and allergic rhinitis, as evidence has shown that these conditions may predispose individuals to develop respiratory infections, and thus subsequently increase use of antibiotics [31]. Furthermore, the timeline between antibiotic exposure and diagnosis was not able to established in our study, increasing the risk of reverse causality. Also, diagnoses of asthma and allergic rhinitis were based on ICD 9 and 10 codes, thus these diseases could have been incorrectly coded in our sample or been missed in mild cases not formally diagnosed with ICD coding. Additionally, the average age of our sample at the time of this study was 5.7 years, and our study may require a relatively older sample in order to accurately capture development of the target childhood diseases. Antibiotic exposure was counted as number of outpatient orders placed in addition to number of times antibiotics were administered in the hospital. Routes of administration, such as oral vs. intravenous, were not distinguished, and thus could have affected exposure levels in our study. Children who received an outpatient antibiotic order may not necessarily have taken the antibiotic as prescribed and/or children may have been prescribed antibiotics from providers outside of the institution that our study was unable to capture, thus potentially skewing the dose-response relationship. Lastly, one of the major challenges in studying the relationship between the gut microbiome and disease development is acknowledging the complex and multifactorial nature of this relationship and controlling for confounding factors. Subsequently, our study controlled for age, race, gender, living in an area of poverty, NICU stay, prematurity, birthweight and delivery method [32, 33]. However, certain exposures, such as environmental factors, maternal age and antibiotic administration, and infant diet were unable to be controlled for due to the nature of data extraction, and thus may have influenced our results. Future steps to expand upon this study would include categorizing antibiotics by class (narrow spectrum and broad spectrum) and waiting for our sample size to grow to capture more disease diagnoses.

## Conclusions

In conclusion, while not indicative of causation, our results suggest that there is a significant positive relationship between early antibiotic administration and the propensity to develop asthma and allergic rhinitis. While the first year of life does not appear to be a sensitive time period for the gut microbiota in regards to allergic rhinitis, it does appear to be important for the development of asthma, and our data further suggests that antibiotic exposure past the first year of life may still have a significant impact on the microbiota and increase the risk of developing future allergic diagnoses. Given these findings, it is plausible that antibiotics may lead to dysbiosis of the pediatric gut microbiota, lending evidence that careful antibiotic stewardship and minimal dosing should be practiced, especially in the pediatric population.

## Data Availability

The datasets used and/or analyzed during the current study are available from the corresponding author on reasonable request.

## Abbreviations

C.I: Confidence Interval
LUMC: Loyola University Medical Center
NH: Non-Hispanic
OR: Odds Ratio
